# Thermally tunable binary-phase VO_2_ metasurfaces for switchable holography and digital encryption

**DOI:** 10.1515/nanoph-2023-0824

**Published:** 2024-02-21

**Authors:** Yuan Liao, Yulong Fan, Dangyuan Lei

**Affiliations:** Department of Materials Science and Engineering, City University of Hong Kong, Kowloon, Hong Kong, China; State Key Laboratory of Optical Technologies on Nano-Fabrication and Micro-Engineering, Institute of Optics and Electronics, Chinese Academy of Sciences, Chengdu 610209, China

**Keywords:** metasurface holography, tunable metasurface, vanadium dioxide, machine learning optimization, information encryption

## Abstract

Metasurface holography has aroused immense interest in producing holographic images with high quality, higher-order diffraction-free, and large viewing angles by using a planar artificial sheet consisting of subwavelength nanostructures. Despite remarkable progress, dynamically tunable metasurface holography in the visible band has rarely been reported due to limited available tuning methods. In this work, we propose and numerically demonstrate a thermally tunable vanadium dioxide (VO_2_) nanofin based binary-phase metasurface, which generates holographic information in the visible varying with temperature. The insulator-to-metal phase transition in VO_2_ nanofins allows two independent binary-phase holograms generated by machine learning to be encoded in the respective phases of VO_2_ and switched under thermal regulation. By elaborately designing the dimensions and compensated phase of VO_2_ nanofins, high-quality images are reconstructed at corresponding temperatures under appropriate chiral illumination. In contrast, much poorer images are produced under inappropriate chiral illumination. We further demonstrate the advantage of applying the VO_2_ phase-compensated metasurface in high-security digital encryption, where two desired character combinations are read out with appropriate excitations and temperatures, whereas one identical fraudulent message is received with inappropriate excitations. Our design approach offers a new and efficient method to realize tunable metasurfaces, which is promisingly adopted in dynamic display, information encryption, optical anti-counterfeiting, etc.

## 1 Introduction

In computational holography, which utilizes the principle of interference and diffraction to calculate and reconstruct the amplitude and phase of light fields [1], holograms (i.e., recorded interference patterns in conventional holography) are computed digitally from predefined images by solving the inverse design problem instead of using optical systems [2], [3]. The Gerchberg–Saxton (GS) algorithm is widely used for phase retrieval in computational holography due to its simplicity and fast convergence, while it usually suffers from limited image quality, stagnating in local optima, and inconsistency in output [4], [5], [6]. Alternatively, the recently fast-developing machine learning-based optimization methods show their advantage in both image quality and computing speed enhancements, especially when designing holograms for three-dimensional display [2], binary-amplitude holograms [7], [8], multilayer holograms [9], [10], etc.

In terms of hologram carriers, broadly used spatial light modulator (SLM) based holograms suffer from modulation errors, high-order diffractions, and narrow viewing angles in their produced holographic images [2]. By contrast, optical metasurfaces are considered as a promising alternative candidate for solving the above problems [11], [12], [13], [14]. By encoding computer-generated holograms into subwavelength unit cells, metasurface holography is capable of generating high-quality and large-viewing-angle holographic images without undesired diffraction orders [15], [16], [17], [18], [19], [20], [21]. Metasurfaces are typically static devices with predefined fixed functionalities. Recently, actively tunable metasurfaces have been experimentally demonstrated with the aid of stretchable substrates [22], [23], [24], electric diodes [25], [26], [27], [28], chemical treatment [29], polarization control [30], liquid crystals [31], multiple-quantum-wells [32], and phase-change materials (PCMs) [33], [34], [35], [36], [37], [38]. However, most PCM-based metasurfaces work in the infrared or gigahertz bands by leveraging different optical behaviors originating from plasmon resonances, whereas their counterparts in the visible are rarely reported.

**Figure 1:**
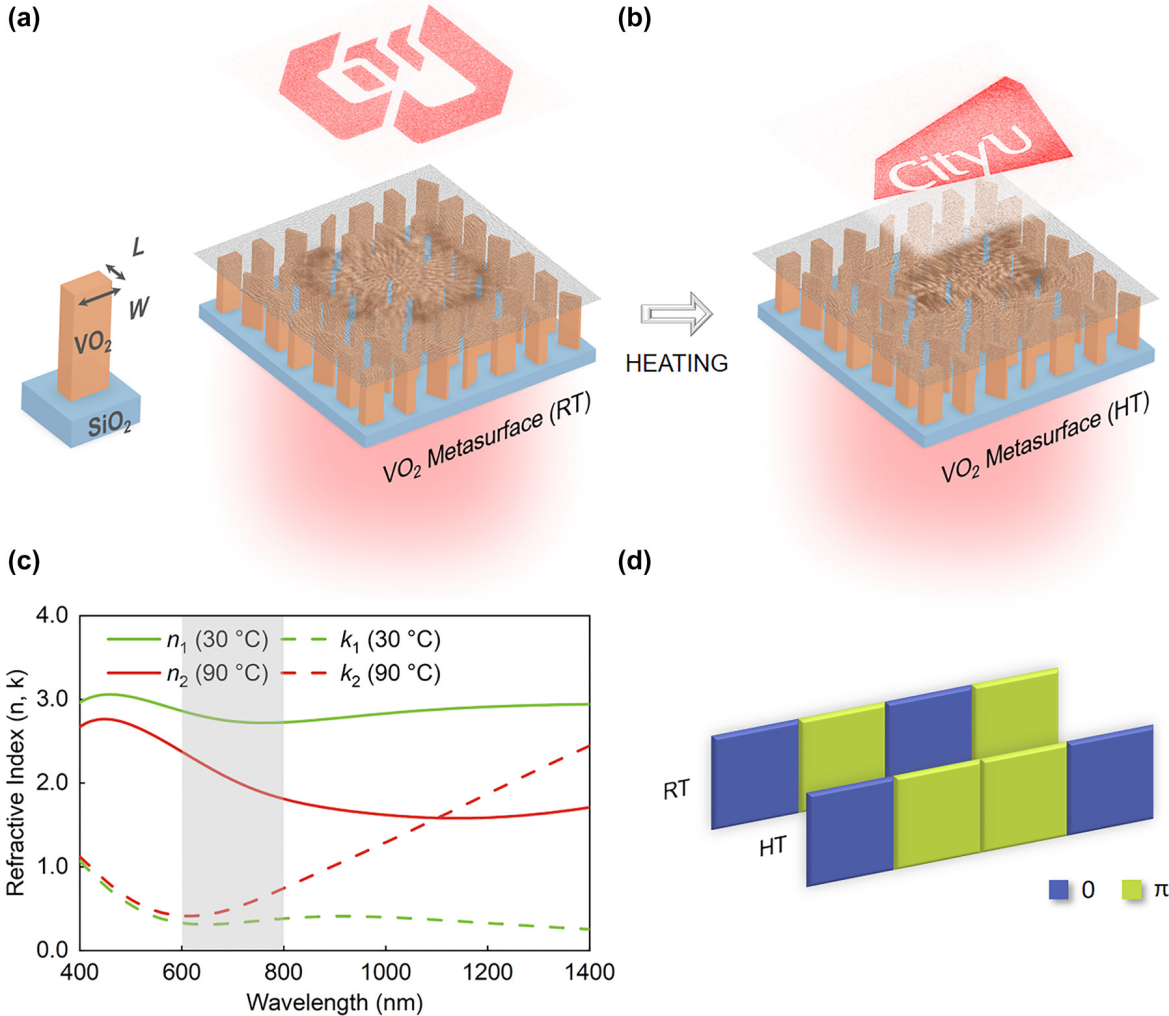
Schematic of thermally tunable VO_2_ metasurface holography and its working principle. (a) and (b) Holographic images generated by the metasurface under illumination at room temperature (RT) (a) and high temperature (HT) (b), respectively. The grayscale in each schematic indicates the phase profile of the metasurface at the corresponding temperature. (c) Experimental dielectric function of a 93 nm thick VO_2_ film at 30 °C (RT) and 90 °C (HT). The shaded area indicates the wavelength range of interest. (d) Working principle of the metasurface realizing switchable binary-phase holograms.

Vanadium dioxide (VO_2_), a volatile temperature-sensitive PCM, has been demonstrated to exhibit the thermally reversible insulator-to-metal phase transition around 68 °C with significant changes in optical properties, leading to various applications of VO_2_ metasurfaces in color generation [39], polarization control [40], [41], perfect absorption [42], etc. In most cases, VO_2_ is integrated into metasurfaces as a thin film underneath nanostructures. The thermally tunable Mie resonances of periodic VO_2_ nanostructures are underexploited [43], [44], [45], [46]. In this work, VO_2_ nanofins are patterned to realize thermally tunable transmissive holographic metasurfaces working at 620 nm and 670 nm, where the refractive index difference between the insulating and metallic phases allows sufficient change in the propagation phase while the extinction coefficients remain relatively low (Figure 1(c)). We develop a gradient descent-based iterative approach to generate two binary-phase (0, π) holograms separately. By elaborately optimizing the unit cells in morphology to fulfill all possible state transitions, i.e., 0 to 0, 0 to π, π to 0, and π to π (Figure 1(d)), the two binary-phase holograms are encoded into the single VO_2_ metasurface and switchable under thermal regulation.

## 2 Methods

### 2.1 Design of unit cells

**Figure 2:**
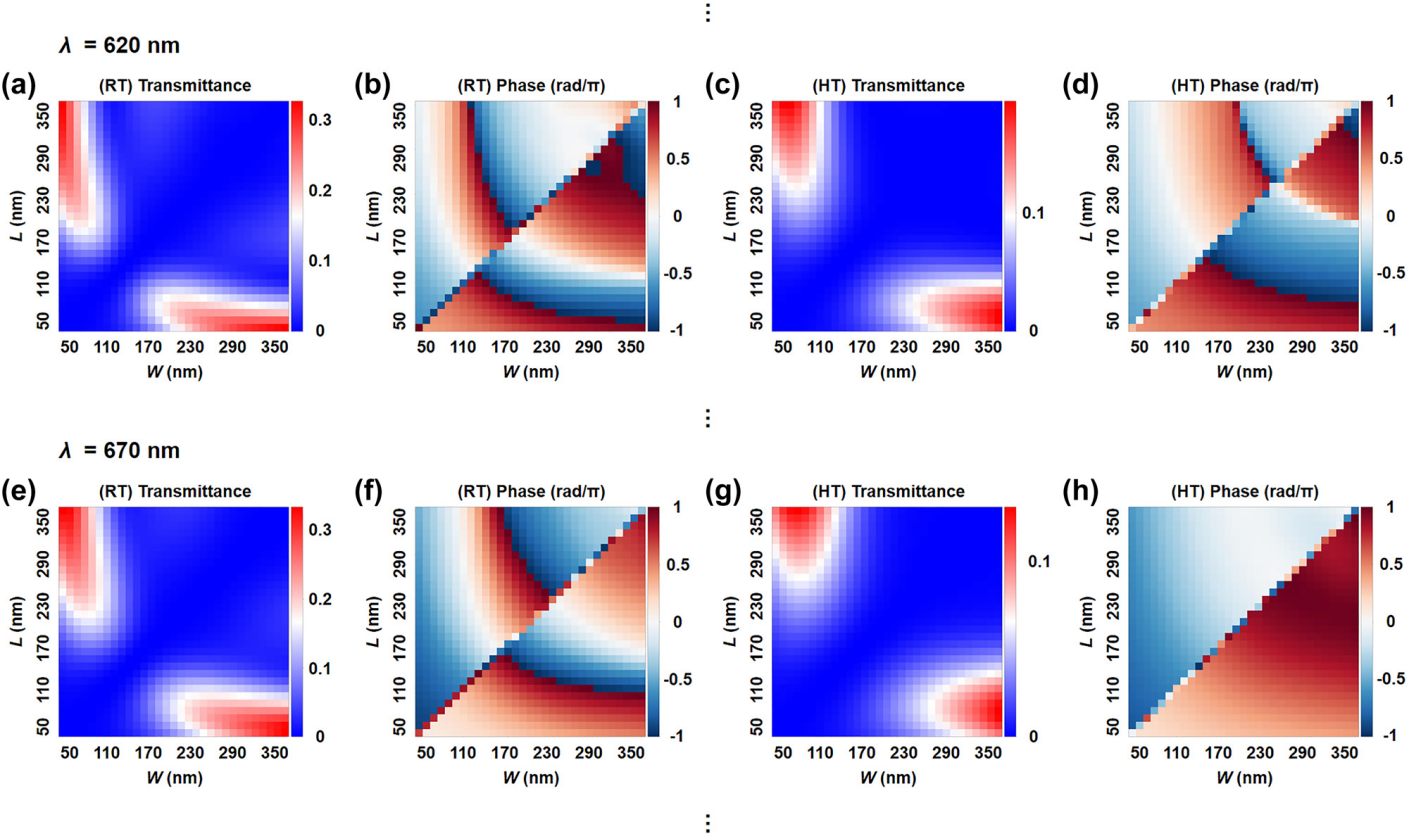
The meta-atom library exemplified at *λ* = 620 nm (a–d) and 670 nm (e–h): cross-polarized light transmittance (a, c, e, g) and propagation phase (b, d, f, h) at RT (a, b, e, f) and HT (c, d, g, h) as a function of *W* and *L* of the nanofin.

The designed optical functionality of the thermally tunable VO_2_ metasurface is illustrated in Figure 1: The older CityU logo shows up when the metasurface is optically excited at room temperature (RT, 30 °C); however, when it works at high temperature (HT, e.g., 90 °C), the latest CityU logo appears. This results in the demand for two independent binary-phase (0, π) profiles and four sets of constituent unit cells in a single metasurface, which need to be engineered to fulfill the four possible state transitions, i.e., 0 to 0, 0 to π, π to 0, and π to π. Therefore, we have a combination of 10 parameters (*T*_RT_ (*i*), *φ*_RT_ (*i*), *T*_HT_ (*i*), *φ*_HT_ (*i*), *φ*_RT_ (*i*) − *φ*_RT_ (*j*), and *φ*_HT_ (*i*) – *φ*_HT_ (*j*)) to consider for one single unit cell, where *T*_R(H)T_ and *φ*_R(H)T_ are the transmittance and propagation phase of cross-polarized light at R(H)T, respectively, and *i*, *j* = 1, 2, 3, or 4 (*i* < *j*) (refer to Tables S1, 2 and the relevant discussion in Supplementary Information for the data searching process). A VO_2_ meta-atom library is then established by performing full-wave finite-element-method simulations (COMSOL Multiphysics 5.5) of VO_2_ nanofins with varying widths and lengths (*W*, *L* from 50 nm to 350 nm) and fixed period *P* = 400 nm and height *H* = 600 nm on a silica substrate. Simulations are performed with VO_2_ refractive index at RT and HT under circularly polarized plane wave excitations ranging from 600 nm to 800 nm, where VO_2_ has sufficiently large refractive index differences between RT and HT and relatively low extinction coefficients [45]. The nanofin morphology-dependent transmittance and propagation phase of meta-atoms exemplified at 620 nm and 670 nm are shown in Figure 2.

**Figure 3:**
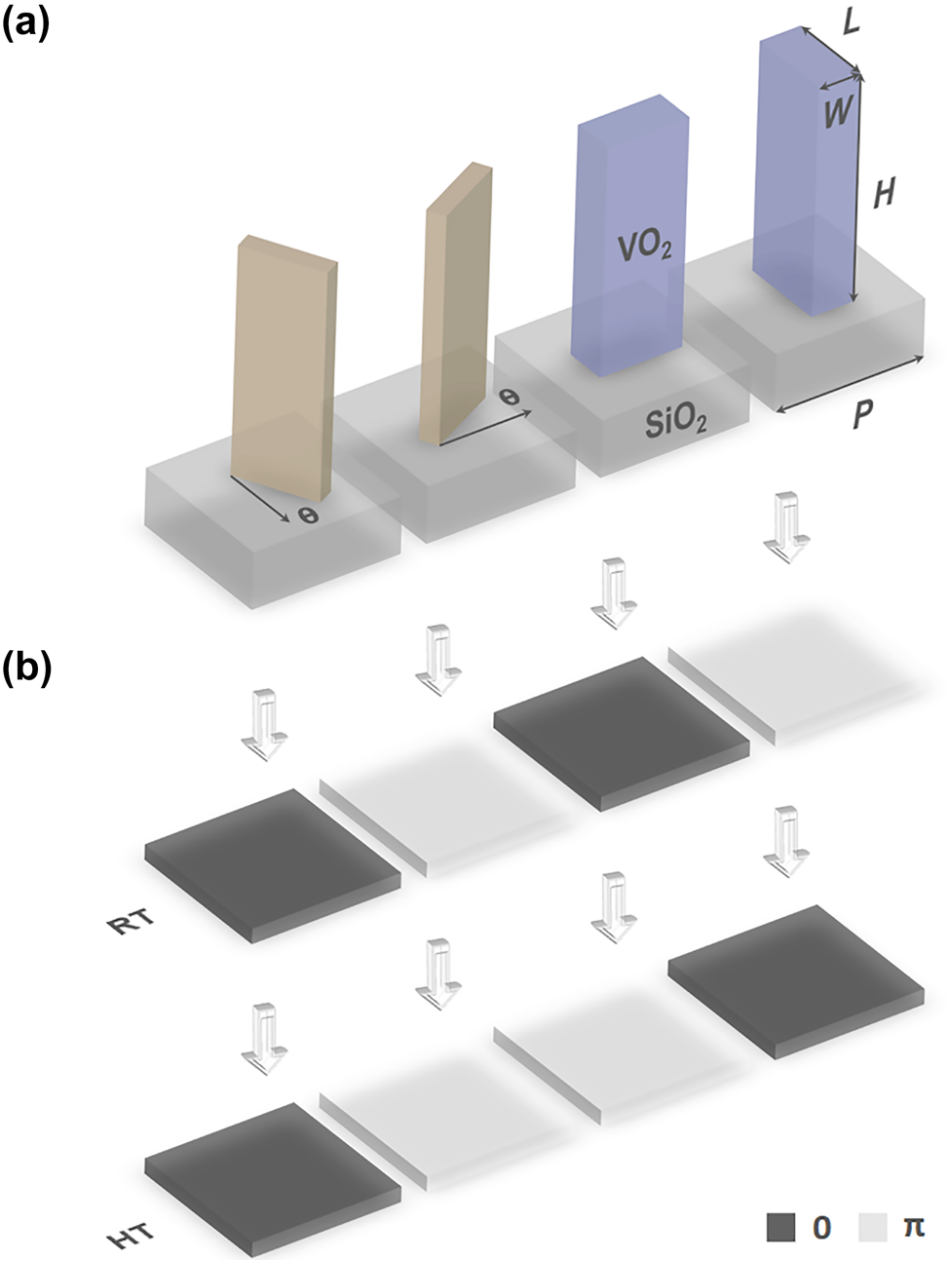
Four selected unit cells as building blocks of the metasurface (a) and their phase states at RT (upper panel) and HT (lower panel) (b).

Unexpectedly, we find that the VO_2_ nanofins with high cross-polarized light transmittance always exhibit limited phase change between RT and HT, as manifested by Figure 2. This necessitates an unavoidable trade-off between high transmittance and high precision in phase difference. Further study reveals that imprecision in phase difference (phase imprecision) has a more pronounced impact on holographic image quality than transmittance imbalance. (see Figures S1 and S2 in Supplementary Information). On this basis, we set the minimum cross-polarized light transmittance to 5 % in the data searching process. On the other hand, it is well known that the Pancharatnam–Berry (PB) phase of a rotated nanofin *φ*_PB_ = ±2*θ* changes its sign with the reversal of incident wave’s chirality, where *θ* is a nanofin’s rotation angle [13]. Therefore, the phase imprecision Δ*φ* can be compensated by rotating the “imperfect” nanofins by *θ* = |0.5Δ*φ*| and reversing the circularity of optical excitation at HT, when the phase imprecisions at RT and HT have the same value with opposed sign. The schematic of the building blocks and the optical properties of the final selected four sets of nanofins with compensation rotation angle *θ* = 0.175π are shown in Figure 3 and Table 1, respectively. The working efficiencies, 14.9 % at RT and 6.0 % at HT, are defined as the arithmetic mean of the cross-polarized light transmittance of nanofins. Dispersion increases the phase imprecision when the illumination wavelength deviates from the designed value. However, the present metasurface can still work within the range of 530 nm–780 nm despite image degradation and decreased working efficiency when working beyond the designed wavelength (Figure S3).

**Table 1:** Selected unit cells with required phase differences and high cross-polarized light transmittance at *λ* = 620 nm. *T*, transmittance; *φ*, propagation phase; and ±2*θ*, additional PB phase. In this case, Δ*φ* = 0.35π at RT, Δ*φ* = −0.35π at HT, and *θ* = 0.175π.

*W* (nm)	*L* (nm)	*T*_RT_ (%)	*φ*_RT_ (rad)	*T*_HT_ (%)	*φ*_HT_ (rad)	State transition
50	220	19.6	0.35π (−2*θ*)	5.9	−0.35π (+2*θ*)	0 ↔ 0
220	110	10.2	0	6.1	π	0 ↔ π
220	50	19.6	1.35π (−2*θ*)	5.9	0.65π (+2*θ*)	π ↔ π
110	220	10.2	π	6.1	0	π ↔ 0

### 2.2 Calculation of binary-phase holograms

**Figure 4:**
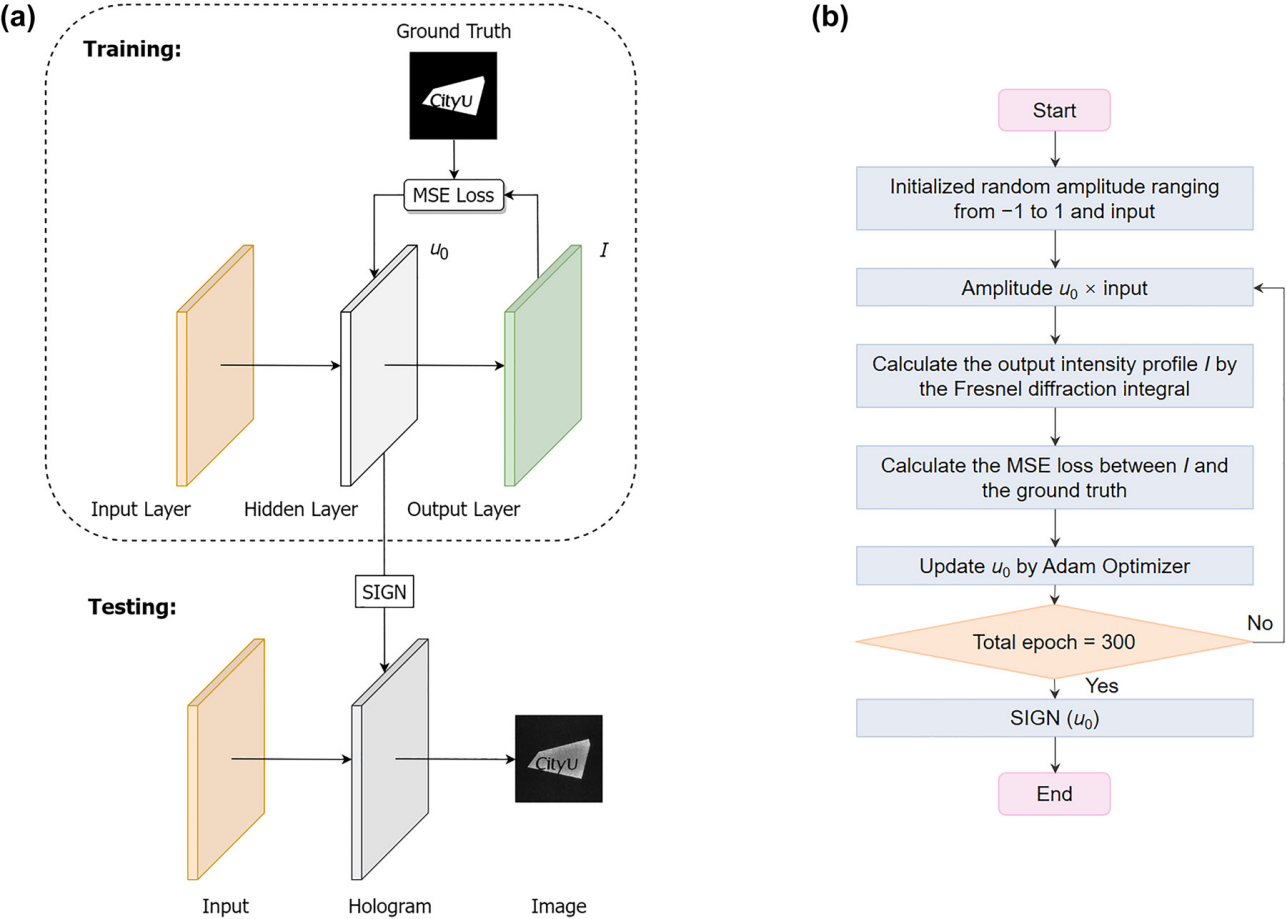
Schematic of the architecture (a) and flowchart (b) of the gradient descent-based iterative approach for hologram calculations. SIGN, sign function.

Images from binary holograms usually suffer from poor quality due to binarization. Inspired by binary neural networks [47], we apply a gradient descent-based iterative approach to generate the binary-phase holograms with 0 and π phases. The architecture and flowchart of the machine learning model are shown in Figure 4. The training model consists of three layers with the size 1000 × 1000: an input layer, a hidden layer, and an output layer, corresponding to incident light, a diffraction plane (i.e., metasurface), and an image plane, respectively. An all-ones matrix representing plane waves serves as the input. Initialized random complex amplitude (real numbers in the range of −1 to 1, where “−” represents a π phase delay) is updated by Adam Optimizer (a conventional stochastic gradient descent optimization algorithm) [48] with a learning rate of 0.01 combined with the mean squared error (MSE) loss function. The forward propagation is calculated on the basis of the Fresnel diffraction theory [49]

(1a) $$I\left(x\text{,}y\right)={\left|F^{-1}\left\{F\left\{u_{0}\left(x_{0}\text{,}y_{0}\right)\right\}\times H\left(f_{x}\text{,}f_{y}\right)\right\}\right|}^{2}\text{,}$$

(1b) $$H\left(f_{x}\text{,}f_{y}\right)={\text{e}}^{\text{i}kz\left[1-\frac{\lambda^{2}}{2}\left(f_{x}^{2}+f_{y}^{2}\right)\right]}\text{,}$$

where *F* and *F* ^−1^ represent the Fourier transform and inverse Fourier transform, respectively; *x*, *y*, and *z* are the spatial coordinates; *x*_0_ and *y*_0_ are the spatial coordinates in the diffraction plane; *f*_*x*_ and *f*_*y*_ are the spatial frequencies; and *u*_0_, *I*, *H*, *λ*, and *k* = 2π/*λ* are the complex amplitude of the source field, intensity of the diffraction field at a distance *z*, transfer function of Fresnel diffraction, wavelength, and wavenumber, respectively. The MSE loss evaluating the difference between the output and the ground truth (i.e., the target image) converges to 0 after 250 epochs of training. The resultant binary-phase profile is taken from the complex amplitude obtained in the last epoch by the sign function. The entire training process lasts for 279 s conducted with the Intel Core i5-1135G7 2.4 GHz CPU and 16 GB of RAM. Instead of binarizing the retrieved phase during each iterative process in the modified GS algorithm (see the simulated reconstructed images from the modified GS algorithm in Figure S4(a) and (c)), all phases in the iterative process are always kept binary, resulting in less loss of quality at the end of the process.

## 3 Results

**Figure 5:**
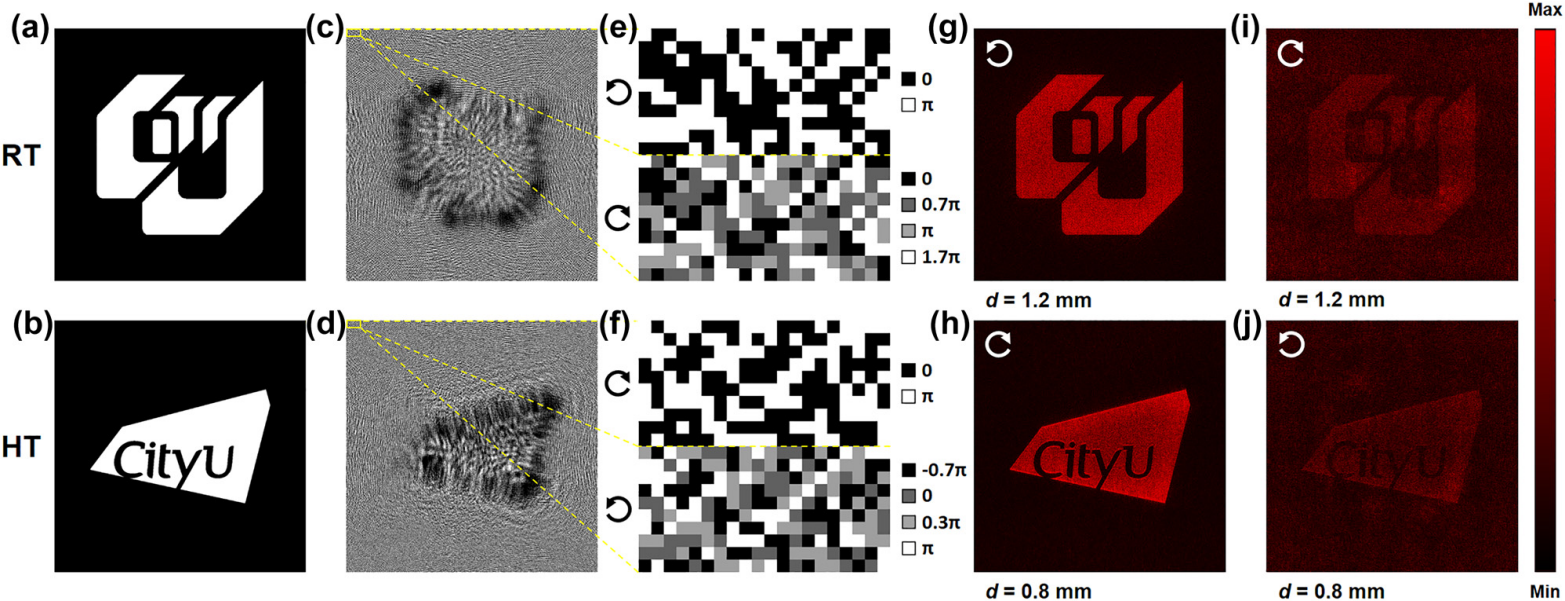
Demonstration of switchable holography. (a) and (b) Target images: binary images of the older version (a) and the latest version (b) of the CityU logo. (c) and (d) Phase profiles of the holograms at RT (c) and HT (d), respectively. (e) and (f) Details of the phase profiles under the illumination with appropriate chirality (top panel) and opposite chirality (bottom panel). (g–j) Holographic images sharing the same color bar (as shown in the rightmost column) under different chiral illumination with PSNRs = 11.38 dB (g), 12.16 dB (h), 7.12 dB (i), and 8.29 dB (j). Circular arrows spinning clockwise and counterclockwise represent RCP and LCP illumination, respectively. *d*, observation distance from the metasurface plane.

The grayscale targeted holographic images at RT and HT are shown in Figure 5(a) and (b), with the machine-learning generated binary-phase profiles consisting of 1000 × 1000 unit cells presented in Figure 5(c) and (d). To avoid holographic image crosstalk, two holographic image planes located 1.2 mm and 0.8 mm from the metasurface plane are purposely assigned to the holograms at RT and HT, respectively. All reconstructed images at 620 nm are calculated using the Fresnel diffraction integral with concrete optical properties of the unit cells listed in Table 1. To highlight the chiral selectivity of phase compensation of 2*θ*, we display in Figure 5(e) and (f) the phase profile of arbitrarily selected 10 × 20 unit cells from Figure 5(c) and (d) under illumination with the appropriate chirality (i.e., left-handed circular polarization (LCP) at RT and right-handed circular polarization (RCP) at HT) in the upper half images and with the opposed chirality in the lower half images. The holograms having desired binary-phase distribution always produce holographic images with sharp edges, high intensity, and high peak signal-to-noise ratios (PSNRs) at two temperatures, as shown in Figure 5(g) and (h). It should be noted that the larger transmittance imbalance between unit cells at RT results in a stronger background and gives rise to a smaller PSNR of Figure 5(g) as compared to Figure 5(h). On the contrary, holographic images created by the optical excitation with opposed chirality display with blunter edges, lower intensity, and lower PSNRs (Figure 5(i) and (j)). Therefore, we infer that all the parameters for generating the holographic images, including the wavelength, polarization, observation distance, and temperature, can be regarded as multiple “keys” in applications such as information encryption and camouflage.

**Figure 6:**
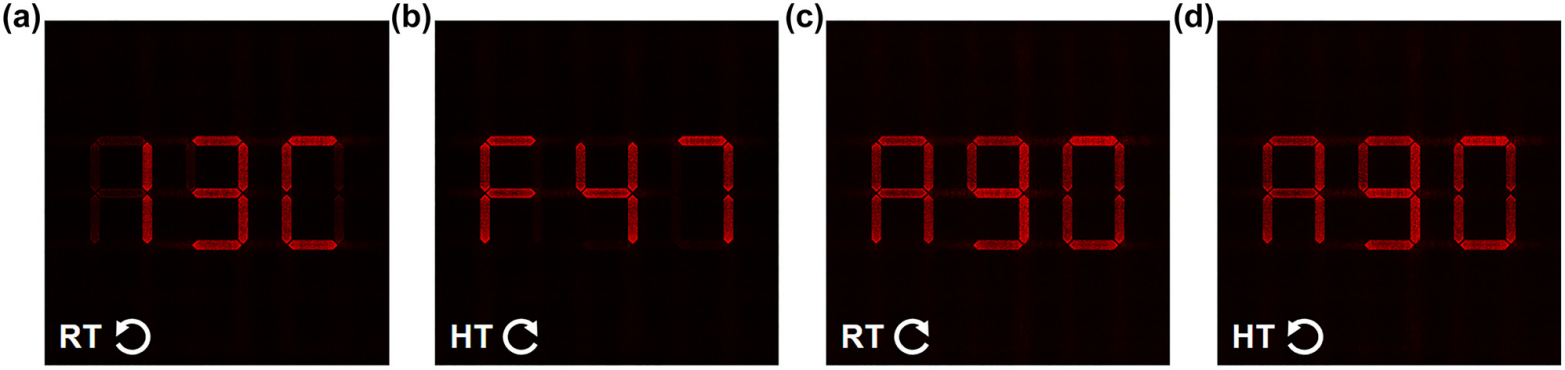
Holographic images produced by the VO_2_ metasurface redesigned for digital encryption under illumination with LCP at RT (a), RCP at HT (b), RCP at RT (c), and LCP at HT (d).

We further demonstrate the superiority of the thermally tunable phase-compensated binary-phase VO_2_ metasurface by showcasing its application in high-security digital display based encryption, where different combinations of numbers and letters are used as the target images in the same image plane. The newly selected wavelength, nanofins’ structures and their optical characteristics are listed in Table 2, where a phase imprecision of Δ*φ* = 0.26π (approaching 0.25π) is exploited. When stimulated by LCP light with a wavelength of 670 nm, the metasurface exhibits a digital display of “13C” (Figure 6(a)). When the chirality of the excitation is reversed at HT, the display changes to “F47” (Figure 6(b)). Conversely, inappropriate chiral excitations result in an identical digital display of “A90” at both RT and HT (Figure 6(c) and (d)). This intriguing application stems from the doubled phase imprecision 2Δ*φ* ≈ π/2, which allows the simultaneous excitation of the two holograms, resulting in an overlapped image of “13C” and “F47”. Based on this scheme, the metasurface could significantly enhance information security in the field of anti-counterfeiting, camouflage, etc.

**Table 2:** New selections of nanofins’ structures with the purposely chosen phase imprecision Δ*φ* = 0.26π at *λ* = 670 nm. *T*, transmittance; *φ*, propagation phase; and ±2*θ*, compensated PB phase with *θ* = 0.13π.

*W* (nm)	*L* (nm)	*T*_RT_ (%)	*φ*_RT_ (rad)	*T*_HT_ (%)	*φ*_HT_ (rad)	State transition
70	340	29.0	0.26π (−2*θ*)	12.0	−0.26π (+2*θ*)	0 ↔ 0
290	140	4.0	0	4.0	π	0 ↔ π
340	70	29.0	1.26π (−2*θ*)	12.0	0.74π (+2*θ*)	π ↔ π
140	290	4.0	π	4.0	0	π ↔ 0

## 4 Perspective

**Figure 7:**
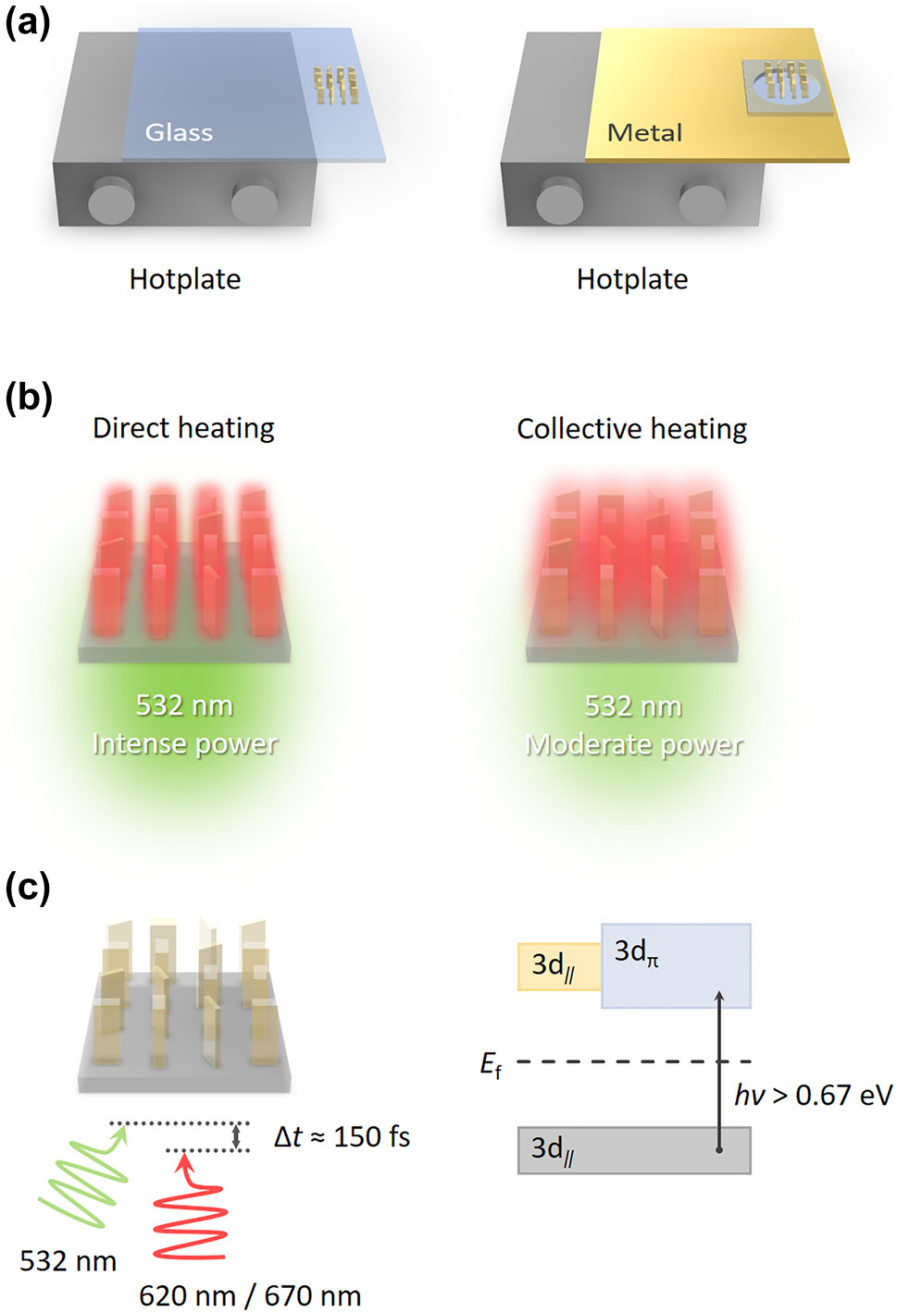
Three typical strategies for triggering the phase transition of VO_2_ nanofins: (a) Bulk heating by using a hotplate with a glass slide supporting the metasurface (left panel). Replacing the glass slide with a metal plate for faster heat conduction (right panel). (b) Photothermal-induced laser heating by a 532 nm CW laser with an intense (left panel) or moderate power density (right panel). The red color in varied level of transparency represents the difference in temperature of VO_2_ nanofins. The laser for imaging is not shown in (a) and (b). (c) Left: ultrafast photoinduced phase transition of VO_2_ nanofins with a pump-probe optical setup, where Δ*t* represents the time delay between the pump and probe pulses. Right: mechanism of the ultrafast phase transition of VO_2_: in the insulating phase, ultrafast photoexcitation of electrons from the 3d*_∥_* valence band to the 3d_π_ conduction band triggers the formation of the metallic phase.

In future experiments, the VO_2_ nanofin based metasurface can be fabricated using state-of-the-art nanofabrication techniques, e.g., electron-beam lithography coupled with dry etching [44], [45], [46]. The phase transition of VO_2_ nanofins can be stimulated in the following ways: (1) Bulk heating has been widely employed for reflection-type VO_2_-based metasurfaces by using a hotplate [44], [50]. Similarly, a hotplate with a glass slide supporting our metasurface can heat up the VO_2_ nanofins to above 68 °C (Figure 7(a)). This method via heat conduction would take tens of minutes to trigger the phase transition due to the bulk heating process [51], [52]. Replacing the glass slide with a metal plate having a hole covered by the metasurface would expedite the heat transfer process (Figure 7(a), right panel) [53], whereas it is still constrained by heating up the glass substrate of the metasurface. (2) Photothermal-induced heating by a continuous-wave (CW) laser can be the second practical method to effectively induce the phase transition in VO_2_ nanofins, especially when the laser’s wavelength is near their absorption peak (see the absorption spectra of the VO_2_ nanofins used in our design in Figure S5 in Supplementary Information). A long-pass filter placed behind the metasurface ensures that the laser for heating will not interfere with imaging experiments. The time required to induce the phase transition of VO_2_ depends on the photothermal management. If the CW laser is intense enough to heat up all VO_2_ nanofins directly to above 68 °C, this process can occur in a shortened time scale, typically from hundreds of picoseconds to a few nanoseconds, due to the nanoscopic dimension of each nanofin (Figure 7(b), left panel) [51], [52], [54]. Otherwise, a collective heating strategy can be employed to achieve the phase transition of VO_2_ under laser illumination with a moderate power density (Figure 7(b), right panel) [53], [55], [56], [57]. In comparison, the time needed to heat up the nanofin ensemble should fall within a timescale of several seconds to minutes [51], [52], depending on the size of the whole metasurface. (3) The fastest and most fascinating method for image switching is based on a pump-probe optical setup. Careful tuning of the time delay between the pump (for photoinduced phase transition of VO_2_) and probe pulses (for imaging) can push the switching cycles towards a (sub-)picosecond timescale (Figure 7(c)) [58], [59].

## 5 Conclusions

In conclusion, we have presented a general approach to designing a thermally tunable binary-phase VO_2_ metasurface. The phase transition characteristics of VO_2_ and the elaborately selected and rotated unit cells enable the metasurface to be switched between two arbitrary binary-phase profiles by thermal tuning. We first introduced the method for encoding two binary-phase holograms into the insulating and metallic phases of a single VO_2_ metasurface. Then, as a proof of concept, we encoded the holograms of two versions of the CityU logo generated by a gradient descent-based iterative approach into the VO_2_ metasurface and read out high-quality holographic images with appropriate circularly polarized excitations. Last, we explored the application of the phase-compensated VO_2_ metasurface in high-security digital display based encryption. Our study demonstrated the possibility of designing thermally tunable VO_2_ metasurfaces in switchable holography and digital encryption in the visible spectrum. More degrees of freedom could be further exploited with multi-level phase difference switching, probably triggering a plethora of applications in dynamic display, optical processing, LiDAR, anti-counterfeiting, high-security encryption/decryption, camouflage, etc. Additionally, our generic design approach could also be readily extended to other actively tunable materials, e.g., germanium antimony tellurium, liquid crystals, and graphene.

## Supplementary Material

Supplementary Material Details
