# Supplementary material for: Thermally tunable binary-phase VO2 metasurfaces for switchable holography and digital encryption
Source: Nanophotonics. 2024 Feb 21;13(7):1109–17. doi: 10.1515/nanoph-2023-0824 (PMC11501404; doi:10.1515/nanoph-2023-0824)
Supplement: Supplementary file 1 — Supplementary Material Details [file j_nanoph-2023-0824_suppl_001.docx]

Supplementary Information for

**Thermally tunable binary-phase VO_2_ metasurfaces for switchable holography and digital encryption**

**Supplementary Note I: Scheme for selecting unit cells from the meta-atom library**

The Method for selecting appropriate unit cells with required phase differences and high cross-polarized light transmittance from the VO_2_ meta-atom library is shown in Table S1, which follows two steps: (1) Select all meta-atoms with cross-polarized light transmittance higher than *T*_min_ and not exceeding *T*_max_ at an arbitrary wavelength in the range of 600 nm to 800 nm. (2) Compare every two meta-atoms selected from the first step, and find pairs with phase differences between −Δ*φ*_max_ and Δ*φ*_max_ at RT and π − Δ*φ*_max_ and π + Δ*φ*_max_ at HT at one wavelength, where Δ*φ*_max_ is the maximum acceptable imprecision in phase differences. After the previous selecting steps, several pairs of unit cells (i.e., meta-atoms) satisfying the state transitions of “0 to 0” and “0 to π” are obtained (“Output of Method” in Table S2). To minimize the impact of imperfection in phase differences and transmittance imbalances, |*φ*_HT_ (*i*) − *φ*_HT_ (*j*) − π| = |*φ*_RT_ (*i*) – *φ*_RT_ (*j*)| (= 2*θ*), *T*_RT_ (*i*) ≈ *T*_RT_ (*j*), and *T*_HT_ (*i*) ≈ *T*_HT_ (*j*) are prioritized in determining the optimal pair. Finally, exchanging the nanofin’s length and width of the selected pair of unit cells obtains another pair satisfying “π to π” and “π to 0” state transitions, which complies with the principle of geometric phase (“Final result” in Table S2). “Output of Method” listing all potential candidates and “Final result” listing the selected ones are given in Table S2.

**Table S1:** Method for selecting appropriate unit cells from the meta-atom library.

| **Method:** Selecting unit cells from the meta-atom library |
| --- |
| **Input:** MAL (*λ*, *W*, *L*, *T*_RT_, *φ*_RT_, *T*_HT_, *φ*_HT_) − Meta-atom library;  *T*_min_ − Allowed minimum of transmittance;  *T*_max_ − Allowed maximum of transmittance;  Δ*φ*_max_ − Allowed maximum imprecision in phase differences (rad/π).  **Initialization:** n − Number of wavelengths × number of meta-atoms;  Set *temp* to empty list;  Set *result* to empty list.  **Output:** Pairs of meta-atoms with required phase differences and transmittance.  **for** *i* = 1 : 1 : n  **if** (*T*_RT_ (*i*) ≥ *T*_min_) **and** (*T*_RT_ (*i*) ≤ *T*_max_) **and** (*T*_HT_ (*i*) ≥ *T*_min_) **and** (*T*_HT_ (*i*) ≤ *T*_max_) **then**  *temp* = [*temp*; MAL (*i*)]  **end if**  **end for**  **for** *j* = 1 : 1 : size (*temp*,1)  **for** *k* = *i* + 1 : 1 : size (*temp*,1)  **if** (*λ* (*j*) = *λ* (*k*)) **and** ((\|*φ*_RT_ (*j*) − *φ*_RT_ (*k*)\| ≤ Δ*φ*_max_) **or** (\|*φ*_RT_ (*j*) − *φ*_RT_ (*k*)\| ≥ 2 − Δ*φ*_max_))  **and** ((\|*φ*_HT_ (*j*) − *φ*_HT_ (*k*)\| ≤ 1 + Δ*φ*_max_) **and** (\|*φ*_HT_ (*j*) − *φ*_HT_ (*k*)\| ≥ 1 − Δ*φ*_max_)) **then**  *result* = [*result*; *temp* (*j*), *temp* (*k*)]  **end if**  **end for**  **end for**  **return** *result* |

**Table S2:** Pairs of unit cells selected by Method with *T*_min_ = 0.059, *T*_max_ = 0.215, and Δ*φ*_max_ = 0.35 (rad/π) and the final result. The phase values of 0.59 and −0.02 are added to the phase shift of the selected unit cells at RT and HT, respectively, as the anormal refraction dictated by the generalized Snell's law is determined by phase gradients rather than absolute phase values. *The phase shift changes by π when *W* and *L* are exchanged.


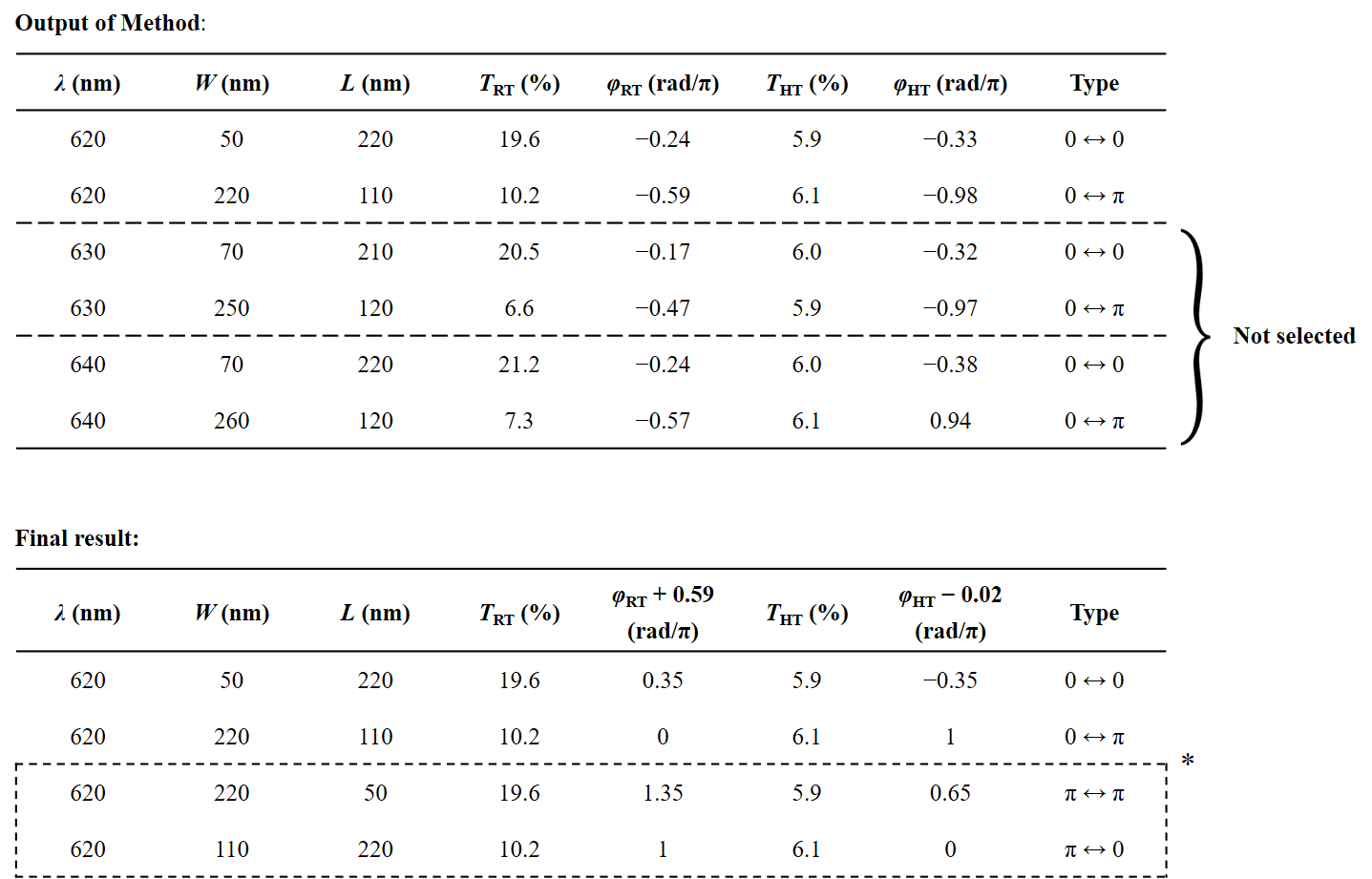


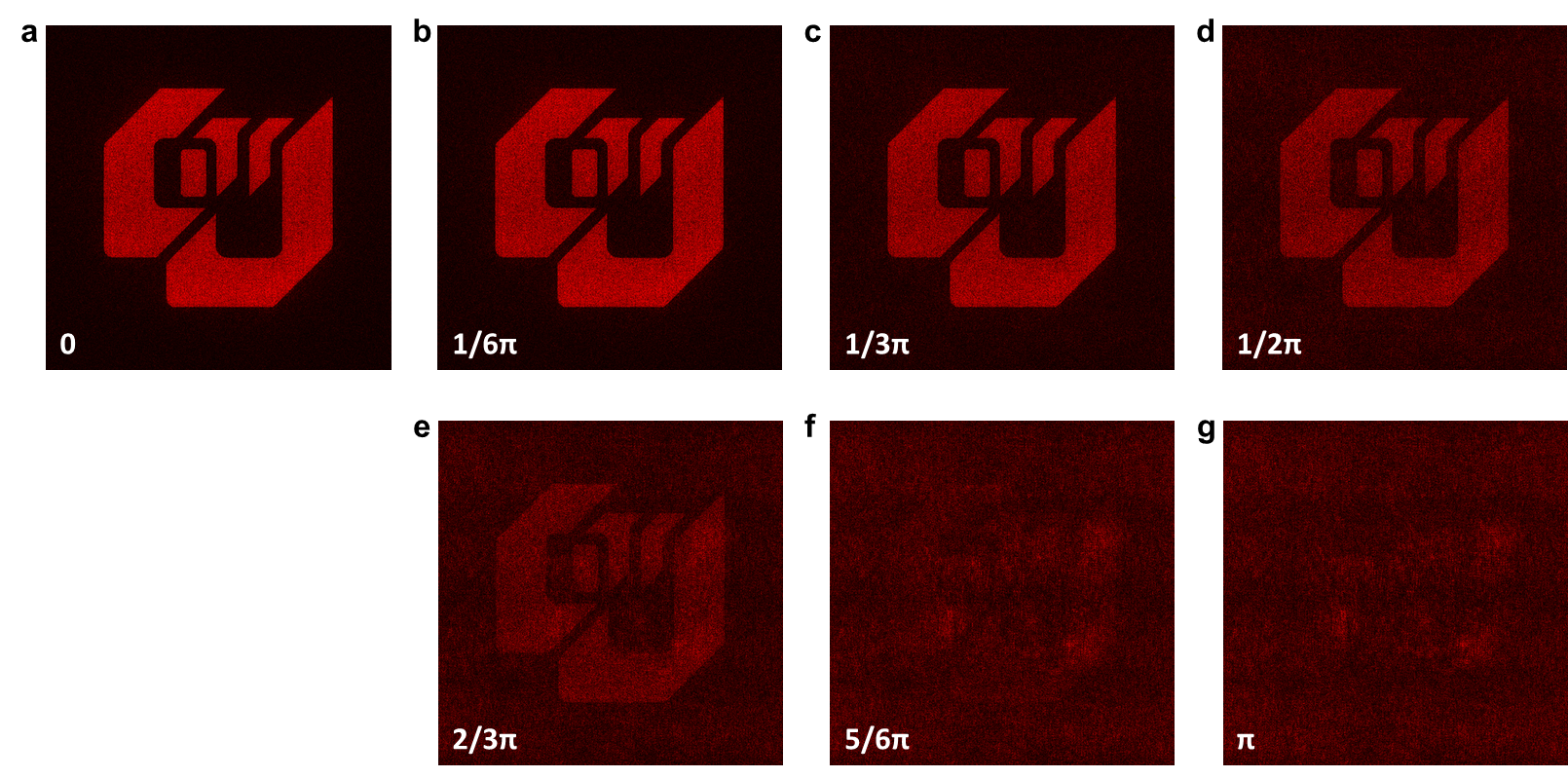


Figure S1: Holographic images with varying phase imprecisions Δ*φ* from 0 to π. Peak signal-to-noise ratios (PSNRs) of the images, 11.53 dB (a), 11.06 dB (b), 9.86 dB (c), 8.43 dB (d), 7.22 dB (e), 6.44 dB (f), and 6.18 dB (g).


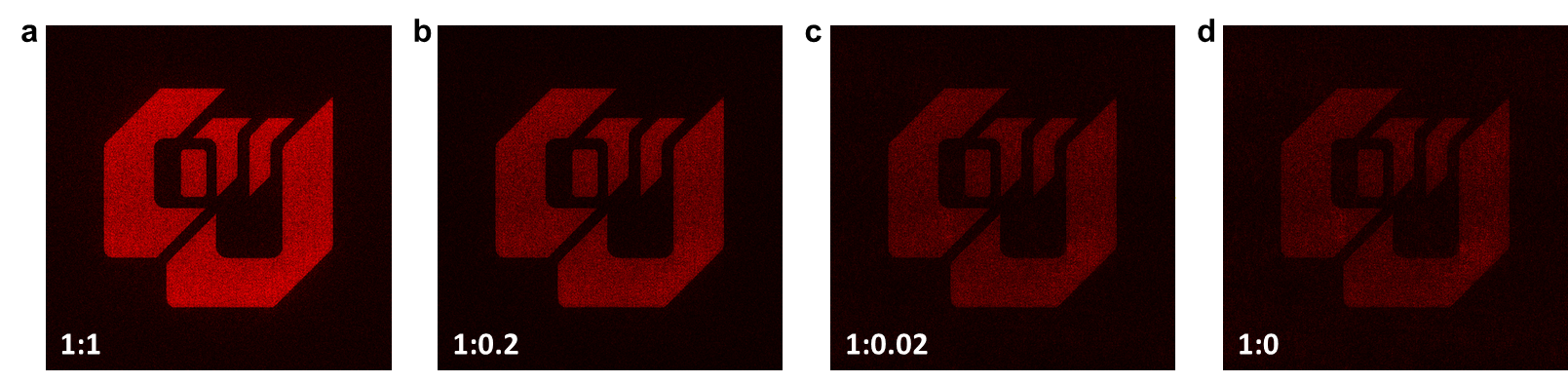


Figure S2: Holographic images with varying transmittance differences from 1:1 to 1:0. PSNRs of the images, 11.53 dB (a), 9.51 dB (b), 8.40 dB (c), and 7.99 dB (d). By comparing Figure S1 and Figure S2, it is evident that phase imprecisions have a more pronounced impact on image quality than transmittance imbalances.


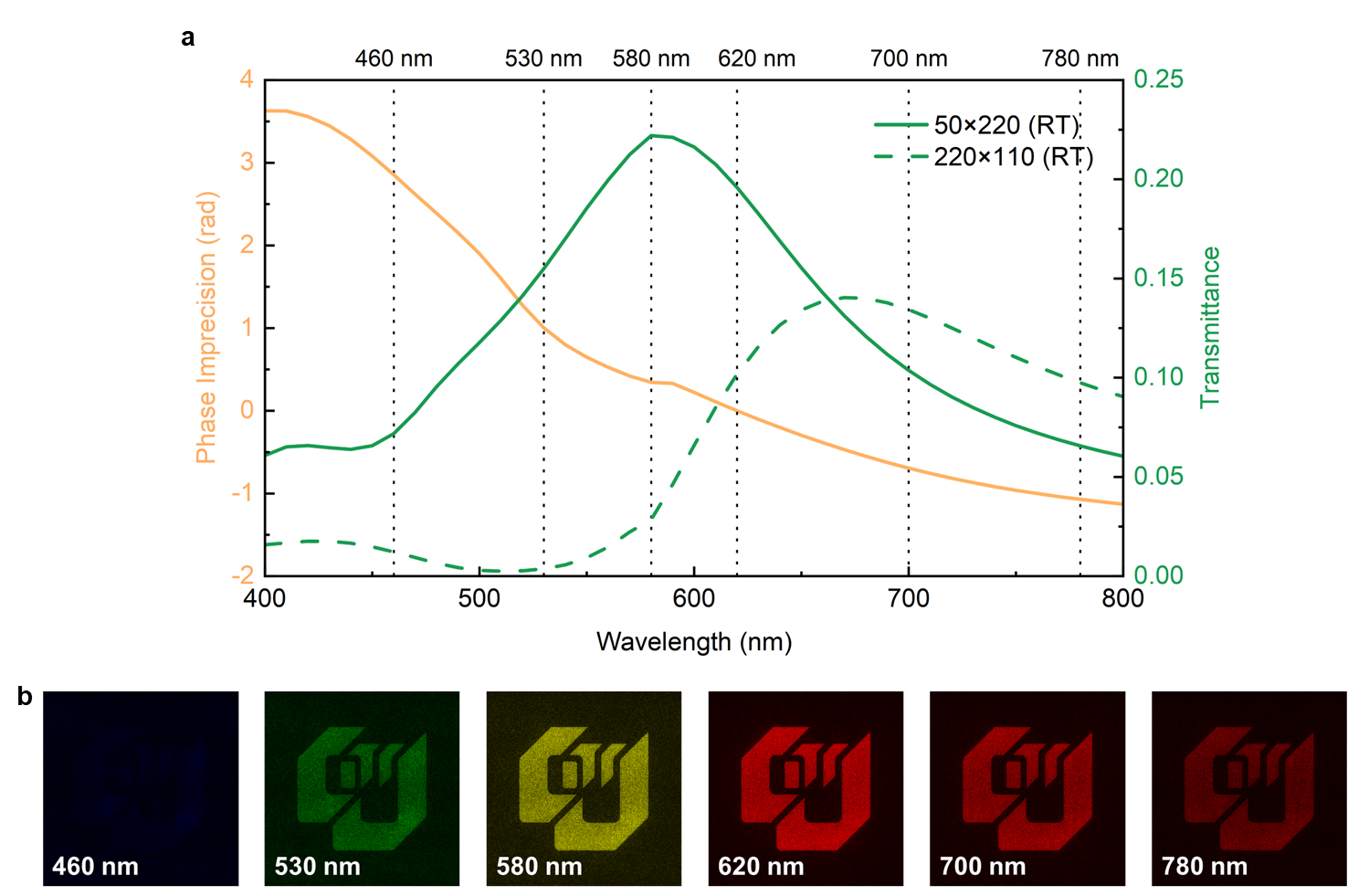


Figure S3: (a) Phase imprecision (orange curve) and transmittance (green curve and green dashed curve) of the VO_2_ nanofins at RT as a function of wavelengths. (b) Reconstructed images at various wavelengths *λ* with PSNR & working efficiency of 6.79 dB & 4.2% (460 nm), 8.31 dB & 7.9% (530 nm), 10.01 dB & 12.5% (580 nm), 11.38 dB & 14.9% (620 nm, designed wavelength *λ*_d_), 10.20 dB & 11.9% (700 nm), and 8.71 dB & 8.2% (780 nm). Observation distance from the metasurface plane, *d* = 1.2 × *λ*_d_ / *λ* (mm).


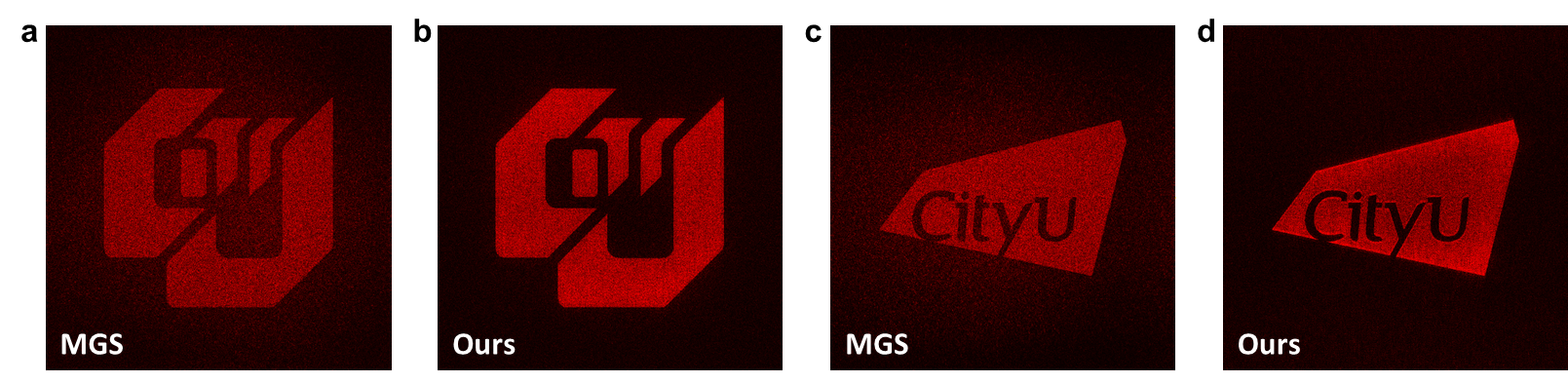


Figure S4: Reconstructed images from the modified GS algorithm (MGS) (a, c) and our approach (b, d). PSNRs of the images, 9.01 dB (a), 11.53 dB (b), 10.75 dB (c), and 12.16 dB (d).


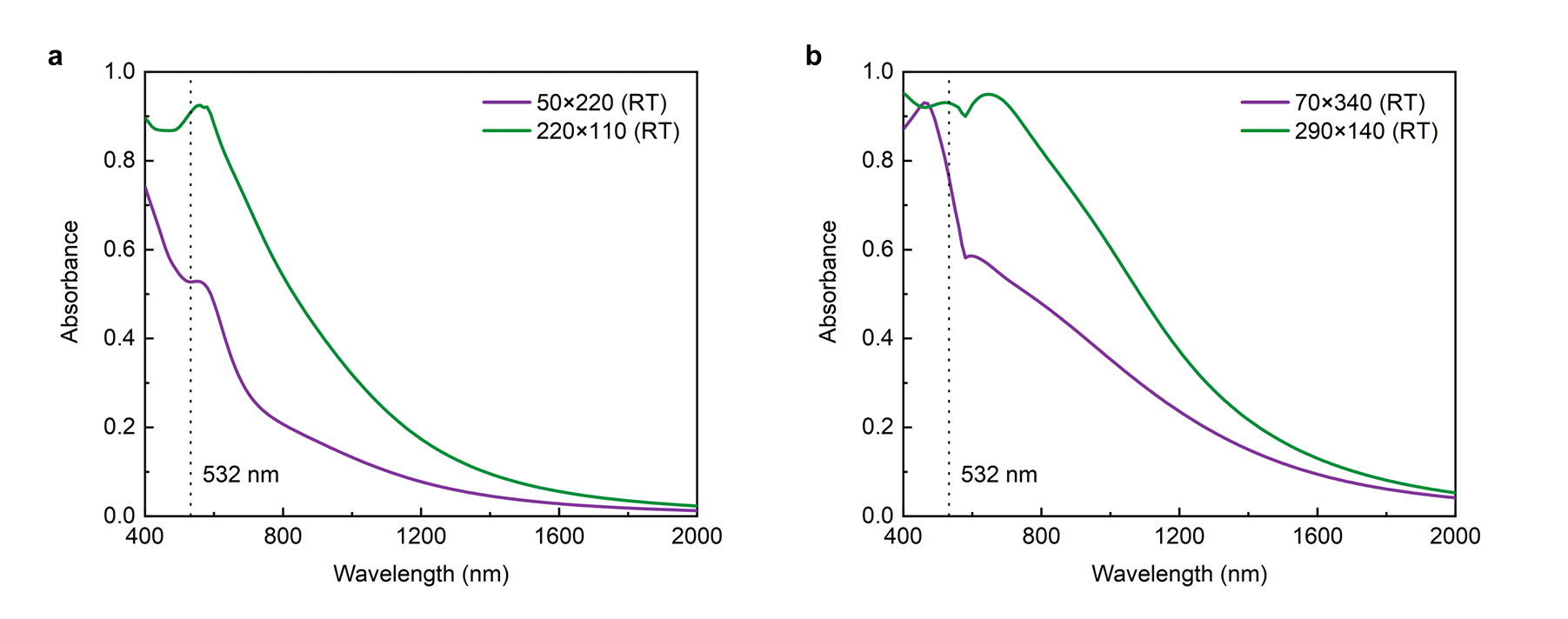


Figure S5: Absorption spectra of the VO_2_ nanofins used in switchable holography (a) and digital encryption (b). A 532 nm CW laser can be used to heat up the VO_2_ nanofins to above 68 °C.
